# Oligodendrocyte Development and Implication in Perinatal White Matter Injury

**DOI:** 10.3389/fncel.2021.764486

**Published:** 2021-11-04

**Authors:** Mahsa Motavaf, Xianhua Piao

**Affiliations:** ^1^Functional Neurosurgery Research Center, Shohada Tajrish Comprehensive Neurosurgical Center of Excellence, Shahid Beheshti University of Medical Sciences, Tehran, Iran; ^2^Eli and Edythe Broad Center of Regeneration Medicine and Stem Cell Research, University of California, San Francisco, San Francisco, CA, United States; ^3^Newborn Brain Research Institute, University of California, San Francisco, San Francisco, CA, United States; ^4^Weill Institute for Neuroscience, University of California, San Francisco, San Francisco, CA, United States; ^5^Division of Neonatology, Department of Pediatrics, University of California, San Francisco, San Francisco, CA, United States

**Keywords:** white matter injury, oligodendrocyte, premature birth, hypoxia-ischemia, myelin

## Abstract

Perinatal white matter injury (WMI) is the most common brain injury in premature infants and can lead to life-long neurological deficits such as cerebral palsy. Preterm birth is typically accompanied by inflammation and hypoxic-ischemic events. Such perinatal insults negatively impact maturation of oligodendrocytes (OLs) and cause myelination failure. At present, no treatment options are clinically available to prevent or cure WMI. Given that arrested OL maturation plays a central role in the etiology of perinatal WMI, an increased interest has emerged regarding the functional restoration of these cells as potential therapeutic strategy. Cell transplantation and promoting endogenous oligodendrocyte function are two potential options to address this major unmet need. In this review, we highlight the underlying pathophysiology of WMI with a specific focus on OL biology and their implication for the development of new therapeutic targets.

## 1. Introduction

Preterm birth, defined as being born before 37 weeks of gestation (gw), is associated with significant adverse neurological outcomes. White matter injury (WMI) refers to myelin deficit in the developing white matter. It is the most common non-hemorrhagic neuropathology in preterm infants, especially in those born before 28 gw (Rantakari et al., 2021). WMI is associated with life-long neurological sequelae, such as cerebral palsy (CP), cognitive delay, and severe motor and sensory impairment.

**FIGURE 1:**
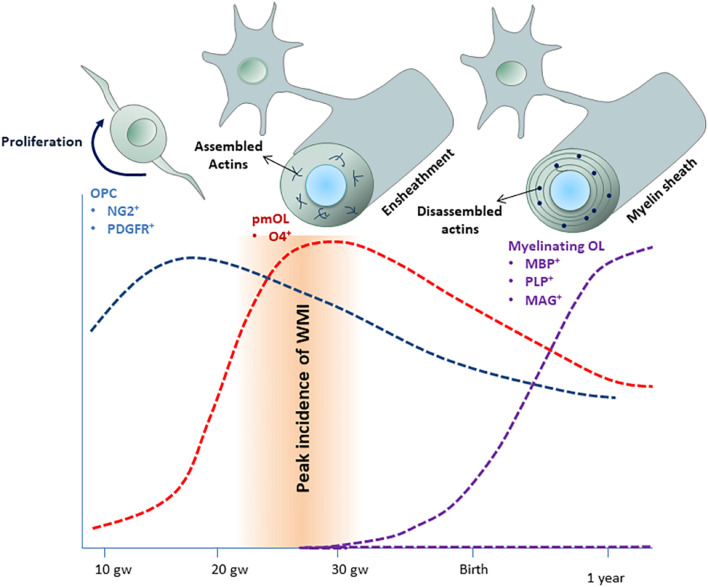
Time-course o f human oligodendroglial cell development. Oligodendrocyte precursor cells (OPCs) appear around 9 gw and expanded between 15 and 20 gw (blue area). pmOLs appear as early as 18 gw but peak between 20 and 30 gw (red area). Myelination starts around 25 gw but mostly occurs during the first year of life and continues for several decades (purple area). The incidence of WMI peaks at 23–32 gw, when pmOLs dominate the OL lineage cells population. Actin filament assembly is essential for ensheathment, whereas dynamic actin filament assembly-disassembly drives myelin sheath growth.

Hypoxic-ischemic injury (HI) and inflammation are two major risk factors leading to WMI (Khwaja and Volpe, 2008; van Tilborg et al., 2018a). Preterm infants spend the first few weeks of their life in neonatal intensive care unit when they are at increased risk for HI and infection. The incidence of WMI peaks at 23–32 gw, a critical window of OL development (Figure 1). During this period, the dominate oligodendrocyte (OL) lineage cells in the developing white matter are O4^+^ premyelinating oligodendrocytes (pmOLs). pmOLs are particularly vulnerable to hypoxic and inflammatory insults (Liu et al., 2013). Limited antioxidant defense mechanisms and high levels of mitochondrial oxygen consumption were proposed as major contributors to their vulnerability (Spaas et al., 2021).

Currently, the management of WMI is limited to supportive measure and symptomatic relief. In this review, we summarize the current understanding of oligodendrogenesis and myelination during normal white matter development, as well as the pathophysiology of WMI. Furthermore, we discuss the current state of experimental therapeutic approaches aiming to restore myelination.

## 2. Oligodendrocyte Development and Central Nervous System Myelination

Myelin, the multilayered glial membrane surrounding axons, is paramount to axonal conductivity and health in the jawed vertebrate nervous system. In addition to enable saltatory and fast conduction of action potentials, myelin supplies axons with energy-rich metabolites such as lactate and pyruvate through the monocarboxylate transporters (Pease-Raissi and Chan, 2021). Importantly, myelination facilitates excitatory presynaptic innervation during development (Wang et al., 2018) as well as learning and memory later in life (Wang et al., 2020). Myelination-enhancing strategies rescues synaptic loss and alleviate cognitive impairments in brains associated white matter pathology (Wang et al., 2018, 2020; Xin and Chan, 2020).

### 2.1 Origin of Oligodendrocytes

In the central nervous system (CNS), myelin is formed by specialized glial cells called OLs, which arise from a lineage-restricted proliferative pool of OL precursor cells (OPCs) (Emery, 2010; van Tilborg et al., 2018b; Elbaz and Popko, 2019). OPCs are derived from neural stem cells (NSCs) in three distinct waves (Kessaris et al., 2006; Rowitch and Kriegstein, 2010). In murine, the initial wave of OPCs is generated from NK2 Homeobox 1 (Nkx2.1)-expressing precursor cells in the medial ganglionic eminence (MGE) and anterior entopeduncular (AEP) regions of ventral telencephalon at embryonic day 12.5 (E12.5) (Kessaris et al., 2006). The second wave emanates from GS homeobox 2 (Gsh2) ^+^ precursors in the lateral ganglionic eminence (LGE) at ∼E15.5 (Kessaris et al., 2006; Chapman et al., 2013). The third wave of OPCs is generated from Empty spiracles homeobox 1 (Emx1)^+^ precursor cells in the cortex around birth (Kessaris et al., 2006); this last wave of OPCs makes up most of OLs in postnatal life. Recent report showed that a subpopulation of first-wave OPCs survives and forms functional cell clusters (Orduz et al., 2019), although biological significance of this finding remains elusive.

In humans, early platelet-derived growth factor receptor α^+^ (PDGFRα^+)^ OPCs emerge in the forebrain at around 10 gw and distribute throughout the developing cerebral cortex during the next few weeks. However, a higher number of OPCs appears only around 15 gw, when they are most numerous in the ganglionic eminences and in the cortical ventricular zone/subventricular zone (Jakovcevski et al., 2009). One characteristic feature of developing human brain is the presence of an enlarged cortical germinal zone called the outer subventricular zone (OSVZ) where outer radial glia (oRG) reside. Although it was originally proposed to exclusively produce neurons, there is compelling evidence indicating that oRG are sources of OLs in later stages of prenatal development (Rash et al., 2019; Huang et al., 2020).

### 2.2 OL Precursor Cells Migration

OPCs migrate to their designated locations under the guidance of a wide variety of mediators, including extracellular chemotropic cues, secreted molecules, and neuronal activity (Simpson and Armstrong, 1999; van Tilborg et al., 2018b; Baydyuk et al., 2020). For instance, glutamate, the main excitatory neurotransmitter released by excitatory neurons, is a putative chemoattractant and stimulates the migration of OPCs through mechanisms that involve AMPA receptor (Mangin et al., 2012). Additionally, spatial gradients of bone morphogenic proteins (BMPs), Sonic hedgehog (Shh), and Wnt proteins determine the direction of migrating OPCs. Remarkably, OPCs use blood vessels as migratory scaffolds to reach their destination in developing CNS by crawling along and/or jumping between vessels (Kurachi et al., 2016; Tsai et al., 2016). Wnt-medicated activation of chemokine receptor CXCR4 in OPCs enables their attraction to the blood vessels presumably via the endothelial-expressed CXCR4 ligand SDF1 (CXCL12) (Tsai et al., 2016).

### 2.3 Oligodendrocyte Proliferation and Differentiation

Once reached to their destined location, OPCs start to proliferate to populate the entire CNS (Hughes et al., 2013). The expansion of OPCs depends on multiple growth factors and motogenic cues, including PDGF, fibroblast growth factor-2 (FGF-2) and insulin-like growth factor-1 (IGF-1). A small population of PDGFRα^+^/NG2^+^-OPCs remains as precursor cells into adulthood, constituting ∼5% of total adult CNS cells. These OPCs also display responsiveness to local CNS injury and differentiate into remyelinating OLs (Dawson et al., 2003; Franklin and Ffrench-Constant, 2008). The majority of OPCs differentiate into mature myelinating OLs through a gradual transition from a proliferative state to an elaboration of cell processes. The timing of OL differentiation is tightly regulated both by cell-intrinsic mechanisms and the extrinsic microenvironment.

OPCs begin to differentiate into pmOLs by losing the progenitor markers (PDGFRα), acquiring a larger cell body, and extending their processes. pmOLs are O4^+^ highly ramified cells that extend their processes to ensheathe axons (Zuchero et al., 2015). The establishment of this glial-axon interaction is a critical point in OL differentiation and mediates target-dependent OLs survival.

Following establishment of primary glial-axon interaction, pmOLs differentiate into mature OLs that are characterized by the expression of galactocerebroside (GalC)/O1 and myelin proteins, such as myelin oligodendrocyte glycoprotein (MOG), myelin basic protein (MBP), myelin associated glycoprotein (MAG), and transmembrane protein proteolipid protein (PLP).

Several transcription factors are involved in the regulation of OL lineage differentiation, among which helix-loop-helix (HLH) family members have been extensively studied. Olig2 acts as a central node to which many pathways converge to drive oligodendrogenesis and maturation (Lu et al., 2002; Ligon et al., 2006). For instance, Olig2 directly induces the expression of SRY-box 10 (SOX10), a well-established regulator involved in OL terminal differentiation and myelin formation. Interaction of SOX10 with several genes such as myelin regulatory factor (MYRF) is critical for full differentiation of OLs. Once induced, MYRF mediates the progression of pmOLs to a mature myelinating state. CF7L2 (Zhao et al., 2016), CHD7 (He et al., 2016), ZFP24 (Elbaz et al., 2018), Hes5, NKX2.2, and NFATC2 are among other factors that cooperate with SOX10 to mediate OL differentiation.

Epigenetic mechanisms including DNA methylation, histone modification, and regulatory non-coding RNAs play permissive roles in OL biogenesis (Tiane et al., 2019; Berry et al., 2020). Histone modifications have been shown to be broadly involved in OPC differentiation (Marin-Husstege et al., 2002; Lyssiotis et al., 2007; Chen et al., 2011; Gregath and Lu, 2018; He et al., 2018). Pharmacological inhibition of histone deacetylases (HDACs), the enzyme family responsible for the removal of acetyl-groups from histones, is showed to be associated with a decrease in OL maturation and differentiation (Marin-Husstege et al., 2002). HDAC inhibition reverses the fate of committed OPCs toward NSC state, suggesting their crucial role during OL development (Lyssiotis et al., 2007).

### 2.4 Central Nervous System Myelination

Once physical interactions between OL and axon occur, the initial layers of myelin rapidly wrap around the axons. Simultaneously, the myelin sheath extends longitudinally along the axon and the myelin membrane layers compact their cytoplasm to form mature myelin. During the myelin sheath growth, actin filaments turnover is the driving force by regulating repetitive cycles of leading edge protrusion and spreading (Nawaz et al., 2015). An individual OL has the capacity to myelinate up to 50 axons, depending on their location within the CNS (Sobottka et al., 2011).

To date, two distinct modes of myelination—axonal activity-dependent vs. independent—have been proposed (Pease-Raissi and Chan, 2021). In activity-dependent myelination, axonal electrical activity and molecular cues such as growth factors and neurotransmitters govern myelination. Both molecular cascades of synaptic and non-synaptic neurotransmission are involved in activity-regulated myelination and remodeling of existing myelin (Almeida et al., 2021). Blocking vesicular mediated neurotransmitter release by tetanus neurotoxin as well as attenuation of neuronal activity reduces percentage of myelinated axons (Suárez et al., 2014; Hines et al., 2015; Koudelka et al., 2016). Furthermore, optogenetic or chemogenetic stimulation of neuronal firing elicits oligodendrogenesis and myelination along the corresponding axons (Gibson et al., 2014; Mitew et al., 2018). Activity-independent myelination is driven and regulated by other factors, including locally secreted factors and axonal diameter (Lee et al., 2013; Bechler et al., 2015).

## 3. Pathophysiology of White Matter Injury

WMI was historically named as periventricular leukomalacia (PVL) (Volpe, 2017). Histologically, PVL begins with focal coagulation necrosis in periventricular white matter and microglial infiltration within hours after the primary insult. This is followed by astrocytic activation several days later, which eventually leads to complete loss of all cellular elements in necrotic areas and cavitation after about 2 weeks (Volpe, 2003, 2017; Hamrick et al., 2004). In severe cases, PVL necrotic foci range from about 1–6 mm in diameter. They can extend into the cerebral cortex and occasionally the subcortical white matter (Back, 2017).

Since 1980’s, the presentation of WMI has changed from cystic PVL to milder diffuse WMI (dWMI), thanks to the advancement of medical technology and improved clinical management of premature babies (Back, 2017; van Tilborg et al., 2018b). Magnetic resonance imaging (MRI) and head ultrasound are used to diagnose WMI. Qualitative abnormalities in WMI, including signal abnormalities in the white matter, ventriculomegaly, and thinning of the corpus callosum are better visualized by MRI (Riddle et al., 2011). The extent and patterns of myelination abnormalities can be variable. Severity and duration of insult as well as the stage of brain maturation likely play a pivotal role in the severity and extent of the WMI (Sosunov et al., 2021).

Preterm birth coincides with the initiation of oligodendrocyte lineage development (Salmaso et al., 2014). During the window of 24–32 gw, the majority of OL lineage cells are early OPCs (NG2^+^/O4^–^) and pmOLs (O4^+^, O1^–^) (Craig et al., 2003; Volpe et al., 2011). pmOLs are particularly vulnerable to insults such as hypoxia and inflammation (Back et al., 1998). Anatomically and functionally immature cerebral vasculature and blood flow autoregulation mechanism contribute to the development of WMI. Furthermore, developmental delay in the expression of antioxidant enzymes in pmOLs is suggested to predispose this specific stage of OL lineage cells to dysfunction or loss (Folkerth et al., 2004; Khwaja and Volpe, 2008).

Examination of human postmortem brains of WMI has revealed dynamic changes in OL lineage cells. In acute lesions, a significant depletion of O4^+^ cells was observed with degenerating O4^+^ cells in the core and intact O4^+^ cells in more superficial zones of the lesions (Back et al., 2005). In subacute injuries, a regenerative response was seen resulting in an expansion of OL progenitor’s pool (Segovia et al., 2008) as well as the total number of OL lineage cells measured as increased Olig2^+^ cell density. Importantly, a significant increase in Olig2^+^ cell density was observed within and immediately adjacent to the necrotic foci but not in the distal areas to the lesions (Billiards et al., 2008; Buser et al., 2012). However, these newly generated progenitors fail to differentiate into mature myelinating OLs (Back and Volpe, 1997; Back et al., 1998, 2002; Billiards et al., 2008; Back, 2017; Sosunov et al., 2021).

The impaired myelination despite the presence of normal or even increased number of Olig2^+^ cells may also in part due to impaired axonal-OL signaling. Indeed, Billiards et al. (2008) showed that significant numbers of OLs express MBP directly in the perikaryon, rather than on the processes, in WMI areas. Dysregulation of MBP mRNA trafficking and/or disruption of oligodendroglial-axonal interaction could be underlying mechanism in failure of myelin sheath formation (Billiards et al., 2008).

Activated astrocytes and microglia contribute to dWMI (Riddle et al., 2011; Buser et al., 2012), as they play both beneficial and detrimental roles in oligodendrogenesis and myelination (Matejuk and Ransohoff, 2020). For instance, STAT3-mediated reactive astrocytes protect myelin development against neuro-inflammation by restricting the aberrant expression of microglial TGFβ-1, an inhibitory factor for OL maturation (Nobuta et al., 2012). In contrast, production of several astrocyte-derived factors (e.g., BMPs, endothelin-1, Jagged1) as well as high molecular weight hyaluronan product, have been shown to inhibit OPC differentiation and myelination (Traiffort et al., 2020). Reactive microglia could disrupt proliferation and differentiation of pmOLs through proinflammatory cytokines, such as tumor necrosis factor alpha (TNFα), interleukin (IL)1β, IL2, and IL17 (Haynes et al., 2003; Steelman and Li, 2011).

## 4. Enhancing Oligodendrocytes Myelination as Therapeutic Strategies Against White Matter Injury

### 4.1 Cell-Based Therapy

Most of our knowledge in restoring CNS myelination with exogenous cells came from preclinical and clinical studies in congenital hypomyelination disorders. Pelizaeus-Merzbacher disease (PMD; OMIM312080) being an exemplar disease for cell-based therapy using various cell sources. PMD is an X-linked disorder caused by mutation in the proteolipid protein-1 (PLP1) gene. It is a progressive congenital disorder of myelin formation, which results in severe neurological disability. There is no effective treatment to date. An open label phase I clinical trial with allogenic human NSCs transplantation was conducted in four individuals with PMD (ClinicalTrials.gov NCT01005004 and NCT01391637) (Gupta et al., 2012, 2019). This study showed a favorable safety profile, long-lasting cell engraftment, and donor-derived myelination (Gupta et al., 2012). At the 2-year post-transplantation follow up, MRI and diffusion tensor imaging (DTI) showed a spectrum of differences between subjects. However, these changes became insignificant at 5-year follow-up (Gupta et al., 2019). On the other hand, the development of donor-specific HLA alloantibodies was detected in two of the four transplanted individuals, suggesting the importance of long-term immunological monitoring (Gupta et al., 2019). The lessons learned from this clinical trial are invaluable for the use of cell-based therapies in demyelinating disease conditions in humans.

In preclinical animal models of hypomyelination disorders, OPCs, NSCs, glial progenitor cells (GPCs), human amnion epithelial cells (hAECs), human umbilical cord blood cells (UCBC), and mesenchymal stem cells (MSCs) have showed beneficial effects in re-establishing myelination and/or function (Potter et al., 2011; Goldman, 2016; Goldman et al., 2021; Table 1). The results from limited human clinical trials have also yielded encouraging results in terms of feasibility, long-term safety, and the therapeutic effect of cell therapy in childhood leukodystrophies and cerebral palsy (Table 2; Wang S. et al., 2013; Goldman, 2017).

**TABLE 1:** Preclinical experiments on cell therapy strategies to restore myelination.

Cell type/source	WMI model	Graft region	Findings	References
GPCs/fetal human	PND0 Shiverer mice	CC and cerebellar peduncle	Improved survival Improved neurological function Functional and progressive donor-derived myelination Formation of normal nodes of Ranvier and transcallosal conduction velocities	Windrem et al., 2008

iPSC-derived OPCs/human	PND0 Shiverer mice	CC	Improved survival Functional and progressive donor-derived myelination	Wang S. et al., 2013

UCBCs/human	0.65 gw fetal sheep/LPS	IV	Attenuation of inflammation Restoration of pmOLs maturation Attenuation of OL death and inflammation Protection of normal white matter development	Paton et al., 2018

Allogeneic UCBCs/fetal sheep	0.7 gw fetal sheep/HI	IV	Attenuation of inflammation and oxidative stress Prevention of OLs loss and Hypomyelination	Li et al., 2016

Allogeneic UCBCs-derived MSCs	0.7 gw fetal sheep/HI	IV	Attenuation of inflammation Maintaining OLs development Protection against hypomyelination	Li et al., 2018

mESCs derived-olig2^+^ cells/Mouse	PND3 rat pups/HI	Left LV	Enhanced myelination Neuroprotective effects Improved neurobehavioral performance	Chen et al., 2015

GRP cells from embryonic spinal cord/Mouse	PND5 mice pups/HI	CC	Reduced long-term survival of GRP cells in WMI model Enhanced myelination Improved neurobehavioral performance	Porambo et al., 2015

Primary NSCs-derived OPCs/second trimester fetal brain tissue	PND3 rat pups/HI	right LV or white matter	Enhanced myelination Reduced structural damage Improved neurobehavioral performance	Wu C. J. et al., 2017

Primary NSCs-derived OPCs/second trimester fetal brain tissue	PND7 rat pups/HI	CV	Attenuation of myelin loss Improved neurobehavioral performance	Kim et al., 2018

Primary NSCs/mice embryos	*In utero* mice embryo/LPS	LV	Alleviated inflammation and gliosis Enhanced myelination in the offspring periventricular region	Borhani-Haghighi et al., 2019

*PND,Postnatal Day; CC, Corpus Callosum; LV, Lateral Ventricle; CV, Cerebral Ventricle.*

**TABLE 2:** Clinical trials of cells therapy for infants and children with CP and childhood leukodystrophies.

Condition	Identifier	Phase/masking	Cell type	Size	Age	Route	Outcomes	References
CP	NCT01404663 NCT01763255	I/Open Label I,II/Open Label	Autologous BM- CD133^+^	12 8	4–12 y	IT	Improved motor and cognitive functions	Zali et al., 2015
	NCT03123562 NCT02569775	II/Open Label	Autologous BMMC	25 40	2–15 y	IT	Improved gross motor function and muscle tone	Nguyen et al., 2017; Thanh et al., 2019
	NCT01147653	II/Quadruple	Autologous UCBC	63	1–6 y	IV	Improved brain connectivity and gross motor function	Sun et al., 2017
	NCT01193660	NA/Quadruple	Allogeneic UCBC + recombinant hEPO	105	10 m–10	IV	Improved motor and cognitive	Min et al., 2013
	NCT01528436	II/Quadruple	Allogeneic UCBC	37	6 m–20 y	IV	Improved muscle strength and gross motor performance	Kang et al., 2015
	NCT01978821	I/Open label	Autologous BM-MSC	52	6 m–15 y	IT + IV	Improved gross motor function	Wang X. et al., 2013

PMD	NCT01005004 NCT01391637	I/Open label	Allogeneic HuCNS-SCs	4	6 m–5 y	FLWM	Durable cell engraftment Donor-specific HLA alloantibodies development Evidence of local donor-derived myelination No conclusive evidence of superior myelination	Gupta et al., 2012, 2019

cALD	NCT00176904 NCT00668564 NCT00383448	II,III/Open label II/Open label II/Open label	Allogeneic HC	135 18 38	2.5–22.3 y	IV	Improved survival Improved functional Disability-free survival in early stage patients with limited cerebral disease at the time of transplantation	Peters et al., 2004; Miller et al., 2011; Pierpont et al., 2017; Raymond et al., 2019

EIKD	NA	Blinded	UCB	19	12–44 d or 142–352 d	IV	Improved lifespan and neurologic outcome in asymptomatic neonates No substantive neurologic improvement after symptoms have developed	Escolar et al., 2005; Wright et al., 2017; Allewelt et al., 2018

*HC, Hematopoietic cell; EIKD, Early Infantile Krabbe Disease; HuCNS-SCs, Human CNS stem cells; cALD, Cerebral adrenoleukodystrophy; BMMC, bone marrow mononuclear cells; FLWM, frontal lobe white matter; y, Year; m, Month; d, Day.*

Despite significant progress, there are major concerns regarding the use of therapeutic cell-based approaches in humans, particularly in non-fatal disorders. The report of tumors developing several years after human fetal brain-derived cell transplantation has heightened anxiety about the potential for neoplasia (Amariglio et al., 2009), in addition to concerns regarding requirements for long-term immunosuppression. Further research is needed to determine the best cell source for these therapeutic approaches.

The goal of cell-based therapy for WMI varies. Some sought to directly protect myelinating cells through immunomodulation/trophic supports and others to functionally replace the damaged cells (Ruff et al., 2013; Li et al., 2018; Rumajogee et al., 2018). In reality, it is likely that transplanted cells exert their beneficial effects through both modes of action. Transplanted OPCs and GPCs in neonatal WMI animal model were able to effectively differentiate into differentiation into OL phenotype. These OLs showed long-term survival (at 2 months post-transplantation) and improved myelination (Porambo et al., 2015; Ogawa et al., 2020).

As inflammation and cellular degeneration play a major role in pathological cascade of WMI, UCBCs with established immunomodulatory, anti-apoptotic, and neurotrophic properties are a promising autologous cell source for WMI cell therapy. Indeed, a number of preclinical and clinical studies have demonstrated that UCBC administration protects white matter development via prevention of OLs loss, restoration of pmOLs maturation, and exhibition of anti-inflammatory and antioxidant functions (Li et al., 2016; Paton et al., 2018; Ren et al., 2020). To date, more than 20 clinical trials for CP treatment using UCB have been registered from clinicaltrials.gov (Table 2).

Cell delivery route influences the engraftment, migration, and distribution of transplanted cells. Intravenous (IV) transplantation is a less invasive method (Min et al., 2013; Cotten et al., 2014; Kang et al., 2015; Sun et al., 2017). However, a number of studies report pulmonary embolisms and accumulation of transplanted cells in undesired peripheral organs (Steiner et al., 2012; Jung et al., 2013; Wu Z. et al., 2017). Other more direct routes of transplantation are intrathecal (IT) and intra-cerebral (IC) (Zali et al., 2015; Nguyen et al., 2017; Thanh et al., 2019). The complexity of brain structure and variable localizations of WMI likely influence the selection of proper transplantation route (Henriques et al., 2019). Further preclinical and clinical studies are needed for the development of optimal administration of cell-based therapy.

### 4.2 Targeting Endogenous Oligodendrocytes

Loss of pmOLs during the acute phase of WMI is followed by a significant increase in these cells, suggesting that OPC deficit may not be the major cause of pathology later in life. Instead, dysregulation of pmOL maturation may be the main mechanism underlying neurologic disability in preterm infants (Buser et al., 2012). Thus, therapeutic enhancement of endogenous oligodendrogenesis and myelination is another promising WMI therapeutic strategy. This can be achieved by either testing known regulators of OL development or high throughput screening (Cayre et al., 2021; Table 3).

**TABLE 3:** Pathways and compounds that have been investigated to enhance endogenous myelination and white matter development in perinatal WMI.

	Pathway/Receptor	Intervention	Action	References
**Target-oriented modulations**	BMP	Noggin	Inhibition	Dizon et al., 2011

	HDAC Sirt1	Sirtinol	Inhibition	Jablonska et al., 2016

	IGF-1	IGF-1 administration	Activation	Guan et al., 2001

	EGF	EGF administration	Activation	Scafidi et al., 2014

	Glutamate	Nbqx	Inhibition	Follett et al., 2000

	Erythropoietin	Erythropoietin therapy	Activation	Fauchère et al., 2015

**Compound identified by screening**	GPR17	Hami3379	Inhibition	Merten et al., 2018

	S1P1	Fingolimod	Activation	Serdar et al., 2016

	GPR56/ADGRG1	3-α-DOG	Activation	Zhu et al., 2019

	PPAR-γ	Pioglitazone	Activation	Yeh et al., 2021

	M1 muscarinic acetylcholine receptor	Clemastine	Inhibition	Cree et al., 2018

	Muscarinic receptor	Benztropine	Inhibition	Deshmukh et al., 2013

	ERK 1/2	Miconazole	Activation	Najm et al., 2015; Su et al., 2018

	Smoothened receptor	Clobetasol	Activation	Najm et al., 2015; Su et al., 2020

	κ-Opioid receptor	U-50488	Inhibition	Mei et al., 2016

	Cholesterol biosynthesis enzymes	Multiple molecules	Inhibition	Hubler et al., 2018; Allimuthu et al., 2019

	Muscarinic receptor	Multiple compounds	Inhibition	Mei et al., 2014; Lariosa-Willingham et al., 2016

	Estrogen receptor	Bazedoxifene	Inhibition/activation	Lariosa-Willingham et al., 2016; Rankin et al., 2019

	Sterol 14-reductase	U-73343	Inhibition	Sax et al., 2021

	Serotonin/norepinephrine transporter adrenergic receptor ion channels	Multiple compounds	Inhibition/activation	Lariosa-Willingham et al., 2016

#### 4.2.1 Testing Known Regulators

Several pathways have been explored for their efficacy in promoting developmental myelin formation in animal models (Table 3), a number of which advanced to human studies. The first is IGF-1, which serves as a major regulator of the proliferation and development of OL lineage (Mason et al., 2003; Cui and Almazan, 2007). IGF-1 was protective in preclinical models of WMI (Guan et al., 2001; Cao et al., 2003; Lin et al., 2009; Cai et al., 2011). Furthermore, there is a positive association between postnatal serum IGF-1 concentration, head circumference, brain volume measures, and developmental scores at 2 years of age (Hansen-Pupp et al., 2011). Clinical trials with IGF-1- binding protein 3 in preterm neonates with a focus on preventing retinopathy of prematurity demonstrated safety profile (ClinicalTrials.gov NCT01096784). Further studies are needed to explore the potential neuroprotective effects of IGF-1 with respect to dWMI (Ley et al., 2013).

Erythropoietin (EPO), originally recognized for its role in erythropoiesis, has also been extensively studied in neurological conditioned (Shingo et al., 2001; Wang et al., 2004; Sola et al., 2005; Iwai et al., 2010). EPO receptors (EPOR) are present in all stages of OL lineage cells. Coordinated expression of EPO and its receptor during CNS development is crucial for the survival of OLs (Ruscher et al., 2002; Knabe et al., 2004; Fan et al., 2011; Kako et al., 2012). Notably, prenatal HI injury disrupts this regulated coordination in ischemia-vulnerable immature OLs, predisposing OLs to apoptosis (Mazur et al., 2010). Thus, administration of EPO provides a potential opportunity to optimize the survival of cells that express EPOR, including OL lineage cells. Indeed, postnatal administration of recombinant human EPO (rhEPO) in animal model of WMI was shown to rescue pmOLs from glutamate-induced excitotoxicity, enhance OL function, promote myelin formation, and improve motor skills (Mazur et al., 2010; Liu et al., 2011; Jantzie et al., 2013). Unfortunately, despite the early encouraging results from human clinical trial (Leuchter et al., 2014; Fauchère et al., 2015; O’Gorman et al., 2015), follow-up study failed to show significant differences in neurodevelopmental outcomes or death (Juul et al., 2020) (ClinicalTrials.gov NCT00413946 and NCT01378273).

#### 4.2.2 High-Throughput Screening and Drug Repurposing

High-throughput screening (HTS) platform allows for the identification of approved compounds for repurposing therapy as well as drug discovery (Eleuteri et al., 2017; Manousi et al., 2021). Indeed, screenings for pharmaceutical compounds that promote myelination have revealed several modulators for G protein-coupled receptor (GPCRs) that are major pharmacological targets for myelin-related diseases (Table 3; Mei et al., 2016; Mogha et al., 2016; Folts et al., 2019).

For instance, HAMI3379, initially developed as a cysteinyl-leukotriene CysLT2 antagonist to treat cardiovascular and inflammatory disorders (Wunder et al., 2010), has the property to enhance OL maturation via antagonizing GPR17 (Merten et al., 2018). Gpr17, which is abundant in pmOLs and undetectable in mature OLs, is a key regulator of OL differentiation.

GPR56/ADGRG1 is an emerging member of the GPCR family with considerable therapeutic potential in neurodevelopmental disorders (Folts et al., 2019). While this multifunctional GPCR is expressed in OPCs, microglia, astrocytes and neurons, cell autonomous function of OPC-specific ADGRG1 is crucial for proper myelination. Strategies to modulate this interaction provide a potential pharmaceutical target for WMI. Indeed, HTS approach targeting GPR56 has revealed 3-α-acetoxydihydrodeoxygedunin (3-α-DOG) and monobodies as GPR56 partial agonists (Stoveken et al., 2018; Zhu et al., 2019; Salzman et al., 2020). Further work is needed to determine their druggable property for WMI.

Fingolimod (FTY720), the first oral drug approved for the treatment of relapsing remitting multiple sclerosis (RRMS), is a functional modulator of Sphingosine 1-phosphate receptor 1 (S1P1). Administration of FTY720 in neonatal model of oxygen-toxicity is reported to attenuate hyperoxia-induced hypomyelination through reduction of hyperoxia-induced oxidative stress and inflammation accompanied with direct protection of developing OLs (Serdar et al., 2016).

Several other drugs and biological compounds, such as Pioglitazone (PPAR-γ agonist) (Yeh et al., 2021), Clemastine (M1 muscarinic acetylcholine receptor antagonist) (Cree et al., 2018), miconazole (ERK 1/2 activator) (Su et al., 2018), clobetasol (Smoothened receptor agonist) (Su et al., 2020) and IDR-1018 (synthetic immunomodulator) (Bolouri et al., 2014) have been identified by HTS to have myelin enhancing property in preclinical and/or clinical trials, although their potential in treating prenatal WMI has not been explored.

#### 4.2.3 Environmental Enrichment and Nutritional Supplementation

The third trimester of pregnancy, during which extreme premature infants are born, is a critical period of neurodevelopment and white matter maturation. The absence of placental nutrients along with low endogenous capacity to synthesize essential biomolecules, particularly in those born extremely preterm, may lead to neurodevelopmental impairment. Indeed, it has been shown that preterm infants have different nutritional needs than term infants (Keunen et al., 2015; Austin et al., 2019). Optimizing early nutritional support for preterm infants has the potential to improve neurodevelopmental outcomes. In this regard, short- and long-chain polyunsaturated fatty acids (PUFAs) as well as cholesterol are indispensable building blocks for myelin production (Dimas et al., 2019; Hussain et al., 2019). Disturbance of cholesterol homeostasis following HI in neonatal brain was associated with worse subcortical white matter development (Kamino et al., 2019; Marangon et al., 2020). To this end, an ongoing clinical trial is currently evaluating the effect of early nutritional supply in brain maturation and neonatal outcomes in preterm infants (ClinicalTrials.gov NCT03555019) (Chan et al., 2016; Wendel et al., 2021).

In addition to optimal early nutrition, the impacts of behavioral interventions and environmental enrichment (EE) have been increasingly appreciated in neurodevelopmental outcomes (Bacmeister et al., 2020; Tooley et al., 2021). Given that the peak of myelination occurs postnatally and continues into early adulthood, environmental enrichment (EE) has attracted major attention as a potential therapeutic strategy in improving neurodevelopmental outcomes (Bacmeister et al., 2020; Tooley et al., 2021). In supporting this notion, preclinical studies showed a reciprocal relationship between motor skill learning and oligodendrogenesis in the motor cortex (McKenzie et al., 2014; Bacmeister et al., 2020). While active myelination is essential for motor skill acquisition, motor learning increases oligodendrogenesis. Encouragingly, it has been demonstrated that early and continuous EE intervention— physical activity, increased socialization, and novel object exposure—attenuated perinatal HI-induced WMI via promotion of oligodendrogenesis and myelination, resulting in functional and behavioral recovery (Forbes et al., 2020). These results support the rationale for using motor skill training to improve myelination.

## Concluding Remarks

Significant progress has been made in our understanding of OL development and myelination. However, more work needs to be done in both pathogenesis and treatment of WMI. Our knowledge on the leading pathophysiology of perinatal WMI remains two-decade old, which is pmOL maturation arrest (Back et al., 2002; Buser et al., 2012; Back, 2017). Single cell RNA sequencing (scRNA-seq) enables molecular characterization of each stages of OL development (Marques et al., 2016). Re-examining pmOLs in normal and WMI brains by scRNAseq may reveal new insights in the development of WMI at molecular level. For example, one important function of pmOLs is to ensheathe axons through F-actin polymerization. Is it possible that HI and neuroinflammation impair this essential developmental process thus leading to pmOL maturation arrest? As for the treatment, there is no effective therapy for WMI despite extensive preclinical efforts. Given the vulnerability of preterm infants and their full life expectancy, any therapeutic modality has to be safe with minimal short- and long-term adverse effect. To fulfill such criteria, UCBC transplantation, EE, and natural compounds derived from breast milk may hold promise in translating to human therapy.

## Author Contributions

MM and XP wrote and revised the manuscript. Both authors contributed to the article and approved the submitted version.

## Conflict of Interest

The authors declare that the research was conducted in the absence of any commercial or financial relationships that could be construed as a potential conflict of interest.

## Publisher’s Note

All claims expressed in this article are solely those of the authors and do not necessarily represent those of their affiliated organizations, or those of the publisher, the editors and the reviewers. Any product that may be evaluated in this article, or claim that may be made by its manufacturer, is not guaranteed or endorsed by the publisher.
